# Supplementary material for: Heparin-based hydrogel scaffolding alters the transcriptomic profile and increases the chemoresistance of MDA-MB-231 triple-negative breast cancer cells
Source: Biomater Sci. 2020 Feb 13;8(10):2786–96. doi: 10.1039/c9bm01481k (PMC7497406; doi:10.1039/c9bm01481k)
Supplement: Supplementary file 2 [file BM-008-C9BM01481K-s002.zip › Supplementary File 4/HP-BhydrogelvControl/Pathways/my_analysis.Gsea.1545201079907/HALLMARK_MTORC1_SIGNALING.html]

Details for gene set HALLMARK\_MTORC1\_SIGNALING[GSEA]

|  || Dataset | expr.class.cls#HYD\_versus\_CONTROL.class.cls#HYD\_versus\_CONTROL\_repos |
| Phenotype | class.cls#HYD\_versus\_CONTROL\_repos |
| Upregulated in class | CONTROL |
| GeneSet | HALLMARK\_MTORC1\_SIGNALING |
| Enrichment Score (ES) | -0.51633275 |
| Normalized Enrichment Score (NES) | -2.4394138 |
| Nominal p-value | 0.0 |
| FDR q-value | 0.0 |
| FWER p-Value | 0.0 |
Table: GSEA Results Summary

  

Fig 1: Enrichment plot: HALLMARK\_MTORC1\_SIGNALING      
 Profile of the Running ES Score & Positions of GeneSet Members on the Rank Ordered List

  

| PROBE | DESCRIPTION (from dataset) | GENE SYMBOL | GENE\_TITLE | RANK IN GENE LIST | RANK METRIC SCORE | RUNNING ES | CORE ENRICHMENT || 1 | PPP1R15A | na |  |  | 1561 | 1.546 | -0.0710 | No |
| 2 | RRP9 | na |  |  | 1722 | 1.481 | -0.0688 | No |
| 3 | CORO1A | na |  |  | 1958 | 1.379 | -0.0713 | No |
| 4 | GSK3B | na |  |  | 2118 | 1.307 | -0.0703 | No |
| 5 | DDIT3 | na |  |  | 2346 | 1.225 | -0.0734 | No |
| 6 | ITGB2 | na |  |  | 2483 | 1.179 | -0.0721 | No |
| 7 | MAP2K3 | na |  |  | 2628 | 1.135 | -0.0716 | No |
| 8 | FDXR | na |  |  | 3632 | 0.886 | -0.1180 | No |
| 9 | DDX39A | na |  |  | 3637 | 0.885 | -0.1118 | No |
| 10 | TOMM40 | na |  |  | 3675 | 0.875 | -0.1075 | No |
| 11 | SLC1A5 | na |  |  | 4248 | 0.762 | -0.1321 | No |
| 12 | NUPR1 | na |  |  | 4351 | 0.741 | -0.1322 | No |
| 13 | MLLT11 | na |  |  | 4837 | 0.651 | -0.1530 | No |
| 14 | LDLR | na |  |  | 5135 | 0.608 | -0.1643 | No |
| 15 | SLC7A5 | na |  |  | 5187 | 0.602 | -0.1626 | No |
| 16 | YKT6 | na |  |  | 5213 | 0.597 | -0.1597 | No |
| 17 | IFI30 | na |  |  | 5258 | 0.592 | -0.1577 | No |
| 18 | CDC25A | na |  |  | 5482 | 0.555 | -0.1655 | No |
| 19 | MCM2 | na |  |  | 5595 | 0.539 | -0.1675 | No |
| 20 | PFKL | na |  |  | 5644 | 0.533 | -0.1662 | No |
| 21 | GGA2 | na |  |  | 5757 | 0.519 | -0.1684 | No |
| 22 | SDF2L1 | na |  |  | 5967 | 0.490 | -0.1759 | No |
| 23 | CCNF | na |  |  | 6105 | 0.470 | -0.1797 | No |
| 24 | ATP2A2 | na |  |  | 6241 | 0.453 | -0.1835 | No |
| 25 | SYTL2 | na |  |  | 6793 | 0.384 | -0.2098 | No |
| 26 | SLC1A4 | na |  |  | 6805 | 0.382 | -0.2076 | No |
| 27 | SLC9A3R1 | na |  |  | 6845 | 0.378 | -0.2069 | No |
| 28 | SQSTM1 | na |  |  | 7012 | 0.359 | -0.2131 | No |
| 29 | EBP | na |  |  | 7038 | 0.355 | -0.2119 | No |
| 30 | IFRD1 | na |  |  | 7053 | 0.353 | -0.2101 | No |
| 31 | FGL2 | na |  |  | 7089 | 0.349 | -0.2094 | No |
| 32 | SLC37A4 | na |  |  | 7261 | 0.331 | -0.2160 | No |
| 33 | PSMC4 | na |  |  | 7347 | 0.321 | -0.2182 | No |
| 34 | NMT1 | na |  |  | 7640 | 0.286 | -0.2315 | No |
| 35 | BTG2 | na |  |  | 7702 | 0.284 | -0.2327 | No |
| 36 | NFKBIB | na |  |  | 7981 | 0.250 | -0.2455 | No |
| 37 | UNG | na |  |  | 8156 | 0.239 | -0.2530 | No |
| 38 | HMBS | na |  |  | 8235 | 0.228 | -0.2554 | No |
| 39 | SLC6A6 | na |  |  | 8243 | 0.227 | -0.2542 | No |
| 40 | ABCF2 | na |  |  | 8730 | 0.178 | -0.2785 | No |
| 41 | MCM4 | na |  |  | 8791 | 0.168 | -0.2804 | No |
| 42 | GCLC | na |  |  | 9105 | 0.140 | -0.2959 | No |
| 43 | ARPC5L | na |  |  | 9236 | 0.124 | -0.3018 | No |
| 44 | FADS2 | na |  |  | 9328 | 0.112 | -0.3058 | No |
| 45 | ACACA | na |  |  | 9410 | 0.104 | -0.3093 | No |
| 46 | TRIB3 | na |  |  | 9434 | 0.099 | -0.3098 | No |
| 47 | ME1 | na |  |  | 9632 | 0.084 | -0.3196 | No |
| 48 | XBP1 | na |  |  | 9718 | 0.073 | -0.3235 | No |
| 49 | RPA1 | na |  |  | 9943 | 0.047 | -0.3350 | No |
| 50 | DHCR7 | na |  |  | 10032 | 0.036 | -0.3393 | No |
| 51 | TUBA4A | na |  |  | 10073 | 0.033 | -0.3412 | No |
| 52 | MTHFD2L | na |  |  | 10368 | 0.010 | -0.3566 | No |
| 53 | QDPR | na |  |  | 10490 | -0.003 | -0.3629 | No |
| 54 | TFRC | na |  |  | 10516 | -0.007 | -0.3642 | No |
| 55 | NFYC | na |  |  | 10534 | -0.008 | -0.3650 | No |
| 56 | CTH | na |  |  | 10798 | -0.037 | -0.3786 | No |
| 57 | PDK1 | na |  |  | 10844 | -0.045 | -0.3807 | No |
| 58 | PLK1 | na |  |  | 10895 | -0.053 | -0.3829 | No |
| 59 | CACYBP | na |  |  | 10963 | -0.060 | -0.3860 | No |
| 60 | TUBG1 | na |  |  | 10996 | -0.065 | -0.3872 | No |
| 61 | INSIG1 | na |  |  | 11030 | -0.071 | -0.3885 | No |
| 62 | GOT1 | na |  |  | 11059 | -0.073 | -0.3894 | No |
| 63 | PDAP1 | na |  |  | 11069 | -0.074 | -0.3893 | No |
| 64 | FADS1 | na |  |  | 11338 | -0.094 | -0.4028 | No |
| 65 | GPI | na |  |  | 11431 | -0.105 | -0.4069 | No |
| 66 | NUFIP1 | na |  |  | 11502 | -0.111 | -0.4097 | No |
| 67 | DHCR24 | na |  |  | 11725 | -0.142 | -0.4204 | No |
| 68 | CDKN1A | na |  |  | 11746 | -0.145 | -0.4204 | No |
| 69 | SERPINH1 | na |  |  | 11747 | -0.145 | -0.4194 | No |
| 70 | PSME3 | na |  |  | 11761 | -0.147 | -0.4190 | No |
| 71 | PNO1 | na |  |  | 11806 | -0.151 | -0.4202 | No |
| 72 | G6PD | na |  |  | 12043 | -0.179 | -0.4314 | No |
| 73 | BUB1 | na |  |  | 12059 | -0.180 | -0.4309 | No |
| 74 | UFM1 | na |  |  | 12108 | -0.188 | -0.4320 | No |
| 75 | HMGCS1 | na |  |  | 12129 | -0.190 | -0.4317 | No |
| 76 | PSMG1 | na |  |  | 12254 | -0.207 | -0.4368 | No |
| 77 | POLR3G | na |  |  | 12329 | -0.217 | -0.4391 | No |
| 78 | CYP51A1 | na |  |  | 12517 | -0.227 | -0.4473 | No |
| 79 | TM7SF2 | na |  |  | 12627 | -0.237 | -0.4513 | No |
| 80 | ACLY | na |  |  | 12653 | -0.241 | -0.4509 | No |
| 81 | PSPH | na |  |  | 12727 | -0.247 | -0.4530 | No |
| 82 | CALR | na |  |  | 12797 | -0.257 | -0.4548 | No |
| 83 | DHFR | na |  |  | 12926 | -0.274 | -0.4596 | No |
| 84 | SLC7A11 | na |  |  | 12949 | -0.278 | -0.4587 | No |
| 85 | RRM2 | na |  |  | 13076 | -0.296 | -0.4632 | No |
| 86 | PIK3R3 | na |  |  | 13096 | -0.298 | -0.4621 | No |
| 87 | SHMT2 | na |  |  | 13483 | -0.351 | -0.4799 | No |
| 88 | BHLHE40 | na |  |  | 13518 | -0.356 | -0.4791 | No |
| 89 | GTF2H1 | na |  |  | 13546 | -0.359 | -0.4779 | No |
| 90 | HMGCR | na |  |  | 13559 | -0.362 | -0.4760 | No |
| 91 | CFP | na |  |  | 13712 | -0.387 | -0.4812 | No |
| 92 | HSPA5 | na |  |  | 13730 | -0.393 | -0.4793 | No |
| 93 | FKBP2 | na |  |  | 13788 | -0.401 | -0.4794 | No |
| 94 | TXNRD1 | na |  |  | 14318 | -0.467 | -0.5039 | No |
| 95 | PSMC6 | na |  |  | 14342 | -0.469 | -0.5017 | No |
| 96 | HSPE1 | na |  |  | 14343 | -0.470 | -0.4984 | No |
| 97 | COPS5 | na |  |  | 14404 | -0.477 | -0.4981 | No |
| 98 | DDIT4 | na |  |  | 14408 | -0.478 | -0.4948 | No |
| 99 | AURKA | na |  |  | 14577 | -0.502 | -0.5000 | No |
| 100 | PSAT1 | na |  |  | 14610 | -0.508 | -0.4981 | No |
| 101 | HSPD1 | na |  |  | 14793 | -0.536 | -0.5038 | No |
| 102 | PSMA3 | na |  |  | 15032 | -0.567 | -0.5123 | Yes |
| 103 | BCAT1 | na |  |  | 15072 | -0.574 | -0.5102 | Yes |
| 104 | SLC2A1 | na |  |  | 15114 | -0.583 | -0.5082 | Yes |
| 105 | PSMD14 | na |  |  | 15161 | -0.591 | -0.5064 | Yes |
| 106 | SCD | na |  |  | 15219 | -0.600 | -0.5051 | Yes |
| 107 | PPA1 | na |  |  | 15233 | -0.603 | -0.5014 | Yes |
| 108 | ALDOA | na |  |  | 15303 | -0.618 | -0.5006 | Yes |
| 109 | LGMN | na |  |  | 15311 | -0.619 | -0.4965 | Yes |
| 110 | PSMA4 | na |  |  | 15398 | -0.634 | -0.4965 | Yes |
| 111 | RPN1 | na |  |  | 15472 | -0.644 | -0.4957 | Yes |
| 112 | TPI1 | na |  |  | 15532 | -0.658 | -0.4941 | Yes |
| 113 | GSR | na |  |  | 15538 | -0.659 | -0.4897 | Yes |
| 114 | EEF1E1 | na |  |  | 15590 | -0.666 | -0.4876 | Yes |
| 115 | ETF1 | na |  |  | 15634 | -0.675 | -0.4850 | Yes |
| 116 | HK2 | na |  |  | 15767 | -0.695 | -0.4869 | Yes |
| 117 | CYB5B | na |  |  | 15952 | -0.733 | -0.4914 | Yes |
| 118 | RIT1 | na |  |  | 16098 | -0.769 | -0.4935 | Yes |
| 119 | ACTR2 | na |  |  | 16139 | -0.775 | -0.4900 | Yes |
| 120 | CCT6A | na |  |  | 16142 | -0.776 | -0.4845 | Yes |
| 121 | EIF2S2 | na |  |  | 16160 | -0.780 | -0.4798 | Yes |
| 122 | STC1 | na |  |  | 16195 | -0.787 | -0.4760 | Yes |
| 123 | NFIL3 | na |  |  | 16230 | -0.795 | -0.4721 | Yes |
| 124 | PNP | na |  |  | 16242 | -0.798 | -0.4669 | Yes |
| 125 | PITPNB | na |  |  | 16254 | -0.799 | -0.4618 | Yes |
| 126 | TCEA1 | na |  |  | 16294 | -0.809 | -0.4580 | Yes |
| 127 | SQLE | na |  |  | 16311 | -0.813 | -0.4530 | Yes |
| 128 | NUP205 | na |  |  | 16374 | -0.827 | -0.4504 | Yes |
| 129 | RDH11 | na |  |  | 16414 | -0.835 | -0.4464 | Yes |
| 130 | IDI1 | na |  |  | 16426 | -0.838 | -0.4410 | Yes |
| 131 | P4HA1 | na |  |  | 16447 | -0.842 | -0.4360 | Yes |
| 132 | FAM129A | na |  |  | 16498 | -0.854 | -0.4325 | Yes |
| 133 | WARS | na |  |  | 16504 | -0.855 | -0.4266 | Yes |
| 134 | PGM1 | na |  |  | 16509 | -0.856 | -0.4207 | Yes |
| 135 | EDEM1 | na |  |  | 16664 | -0.889 | -0.4224 | Yes |
| 136 | GMPS | na |  |  | 16723 | -0.905 | -0.4190 | Yes |
| 137 | UBE2D3 | na |  |  | 16767 | -0.917 | -0.4147 | Yes |
| 138 | PSMC2 | na |  |  | 16782 | -0.922 | -0.4088 | Yes |
| 139 | PRDX1 | na |  |  | 16800 | -0.928 | -0.4030 | Yes |
| 140 | PHGDH | na |  |  | 16845 | -0.939 | -0.3986 | Yes |
| 141 | STARD4 | na |  |  | 16852 | -0.942 | -0.3922 | Yes |
| 142 | GLA | na |  |  | 16858 | -0.944 | -0.3857 | Yes |
| 143 | SLC2A3 | na |  |  | 16869 | -0.946 | -0.3794 | Yes |
| 144 | HSPA4 | na |  |  | 16886 | -0.951 | -0.3734 | Yes |
| 145 | UCHL5 | na |  |  | 16916 | -0.961 | -0.3681 | Yes |
| 146 | ADIPOR2 | na |  |  | 16920 | -0.962 | -0.3613 | Yes |
| 147 | STIP1 | na |  |  | 16929 | -0.964 | -0.3548 | Yes |
| 148 | PSMB5 | na |  |  | 16936 | -0.966 | -0.3482 | Yes |
| 149 | MTHFD2 | na |  |  | 16986 | -0.982 | -0.3437 | Yes |
| 150 | ACSL3 | na |  |  | 17071 | -1.007 | -0.3409 | Yes |
| 151 | ATP5G1 | na |  |  | 17278 | -1.076 | -0.3440 | Yes |
| 152 | ADD3 | na |  |  | 17325 | -1.093 | -0.3386 | Yes |
| 153 | PSMD12 | na |  |  | 17327 | -1.093 | -0.3308 | Yes |
| 154 | PLOD2 | na |  |  | 17330 | -1.095 | -0.3231 | Yes |
| 155 | HSPA9 | na |  |  | 17460 | -1.137 | -0.3217 | Yes |
| 156 | VLDLR | na |  |  | 17499 | -1.153 | -0.3155 | Yes |
| 157 | ELOVL6 | na |  |  | 17624 | -1.209 | -0.3133 | Yes |
| 158 | ATP6V1D | na |  |  | 17625 | -1.209 | -0.3046 | Yes |
| 159 | SERP1 | na |  |  | 17632 | -1.213 | -0.2962 | Yes |
| 160 | SEC11A | na |  |  | 17640 | -1.217 | -0.2879 | Yes |
| 161 | M6PR | na |  |  | 17650 | -1.221 | -0.2796 | Yes |
| 162 | IMMT | na |  |  | 17677 | -1.232 | -0.2721 | Yes |
| 163 | TMEM97 | na |  |  | 17777 | -1.277 | -0.2682 | Yes |
| 164 | CTSC | na |  |  | 17837 | -1.302 | -0.2619 | Yes |
| 165 | TES | na |  |  | 17844 | -1.305 | -0.2529 | Yes |
| 166 | EGLN3 | na |  |  | 18026 | -1.399 | -0.2524 | Yes |
| 167 | PSMD13 | na |  |  | 18042 | -1.410 | -0.2430 | Yes |
| 168 | HSP90B1 | na |  |  | 18044 | -1.410 | -0.2330 | Yes |
| 169 | IDH1 | na |  |  | 18161 | -1.475 | -0.2285 | Yes |
| 170 | ASNS | na |  |  | 18216 | -1.509 | -0.2205 | Yes |
| 171 | AK4 | na |  |  | 18295 | -1.561 | -0.2134 | Yes |
| 172 | NAMPT | na |  |  | 18342 | -1.596 | -0.2044 | Yes |
| 173 | TBK1 | na |  |  | 18386 | -1.630 | -0.1950 | Yes |
| 174 | USO1 | na |  |  | 18436 | -1.663 | -0.1856 | Yes |
| 175 | SRD5A1 | na |  |  | 18457 | -1.677 | -0.1746 | Yes |
| 176 | ACTR3 | na |  |  | 18460 | -1.682 | -0.1627 | Yes |
| 177 | GLRX | na |  |  | 18498 | -1.703 | -0.1524 | Yes |
| 178 | ELOVL5 | na |  |  | 18504 | -1.707 | -0.1404 | Yes |
| 179 | LTA4H | na |  |  | 18589 | -1.771 | -0.1321 | Yes |
| 180 | SSR1 | na |  |  | 18609 | -1.786 | -0.1203 | Yes |
| 181 | GBE1 | na |  |  | 18637 | -1.827 | -0.1086 | Yes |
| 182 | EPRS | na |  |  | 18657 | -1.855 | -0.0963 | Yes |
| 183 | RAB1A | na |  |  | 18690 | -1.887 | -0.0845 | Yes |
| 184 | CXCR4 | na |  |  | 18827 | -2.050 | -0.0769 | Yes |
| 185 | CD9 | na |  |  | 18879 | -2.138 | -0.0643 | Yes |
| 186 | PGK1 | na |  |  | 18894 | -2.154 | -0.0495 | Yes |
| 187 | SKAP2 | na |  |  | 18896 | -2.162 | -0.0341 | Yes |
| 188 | HPRT1 | na |  |  | 18898 | -2.164 | -0.0186 | Yes |
| 189 | SORD | na |  |  | 18930 | -2.228 | -0.0043 | Yes |
| 190 | CCNG1 | na |  |  | 19060 | -2.553 | 0.0073 | Yes |
Table: GSEA details [plain text format]

  

Fig 2: HALLMARK\_MTORC1\_SIGNALING      
 Blue-Pink O' Gram in the Space of the Analyzed GeneSet

  

Fig 3: HALLMARK\_MTORC1\_SIGNALING: Random ES distribution      
 Gene set null distribution of ES for **HALLMARK\_MTORC1\_SIGNALING**
